# PTK 7 Is a Transforming Gene and Prognostic Marker for Breast Cancer and Nodal Metastasis Involvement

**DOI:** 10.1371/journal.pone.0084472

**Published:** 2014-01-07

**Authors:** Silvia Gärtner, Angela Gunesch, Tatiana Knyazeva, Petra Wolf, Bernhard Högel, Wolfgang Eiermann, Axel Ullrich, Pjotr Knyazev, Beyhan Ataseven

**Affiliations:** 1 Department of Molecular Biology, Max-Planck-Institute of Biochemistry, Martinsried, Germany; 2 Department of Gynecology and Obstetrics, Red Cross Hospital, Munich, Germany; 3 Department of Gynecology and Gynecologic Oncology, Kliniken Essen-Mitte, Evangelische Huyssens-Stiftung, Essen, Germany; 4 Department of Pathology, Red Cross Hospital, Munich, Germany; 5 Institute for medical statistics and epidemiology, Klinikum rechts der Isar, Technical University, Munich, Germany; 6 Department of Gynecology and Gynecologic Oncology, Interdisziplinäres Onkologisches Zentrum, Munich, Germany; Stony Brook University, United States of America

## Abstract

Protein Tyrosin Kinase 7 (PTK7) is upregulated in several human cancers; however, its clinical implication in breast cancer (BC) and lymph node (LN) is still unclear. In order to investigate the function of PTK7 in mediating BC cell motility and invasivity, PTK7 expression in BC cell lines was determined. PTK7 signaling in highly invasive breast cancer cells was inhibited by a dominant-negative PTK7 mutant, an antibody against the extracellular domain of PTK7, and siRNA knockdown of PTK7. This resulted in decreased motility and invasivity of BC cells. We further examined PTK7 expression in BC and LN tissue of 128 BC patients by RT-PCR and its correlation with BC related genes like HER2, HER3, PAI1, MMP1, K19, and CD44. Expression profiling in BC cell lines and primary tumors showed association of PTK7 with ER/PR/HER2-negative (TNBC-triple negative BC) cancer. Oncomine data analysis confirmed this observation and classified PTK7 in a cluster with genes associated with agressive behavior of primary BC. Furthermore PTK7 expression was significantly different with respect to tumor size (ANOVA, p = 0.033) in BC and nodal involvement (ANOVA, p = 0.007) in LN. PTK7 expression in metastatic LN was related to shorter DFS (Cox Regression, p = 0.041). Our observations confirmed the transforming potential of PTK7, as well as its involvement in motility and invasivity of BC cells. PTK7 is highly expressed in TNBC cell lines. It represents a novel prognostic marker for BC patients and has potential therapeutic significance.

## Introduction

Breast cancer (BC) is the most commonly diagnosed malignoma and the leading cause of cancer related death in women worldwide [1]. The utilization of clinicopathological and tumor molecular characteristics to determine patient prognosis and response to treatment are important features in the current management of BC. Recognized prognostic factors predicting disease outcome include tumor grade and size, hormone receptor status, HER2 expression, lymph node status, and patient age [2]. Although these classical prognostic markers are reliable in general, more specific prognostic and predictive markers are needed. This requires that we have a better understanding of the molecular mechanism of BC development and metastazation.

The receptor protein tyrosine kinase PTK7, also known as CCK4, was discovered as a gene overexpressed in colon cancer cell lines [3]. PTK7 is characterized as a transmembrane glycoprotein of 1071 amino acids containing an extracellular domain with seven immunoglobulin (Ig)-like loops and a catalytically inactive tyrosine kinase domain [4], [5]. The human PTK7 gene is located on chromosome 6 (6p21.1–p12.2) and consists of 20 exons [6]. It was recently shown that the orphan receptor PTK7 plays a major role in non-canonical Wnt/planar cell polarity (PCP) signaling during vertebrate neural crest movement and establishment of inner ear hair cell polarity [7]. Perhaps the function of PTK7 is not only limited to PCP. An interaction with factors in canonical Wnt signaling e.g. β-catenin is discussed, but the proper role of PTK7 is still controversial. Puppo et al [8] suggested that PTK7 seems to favour β-catenin stabilization and canonical Wnt signaling, whereas Peradziryi et al [9] propose an inhibition of these pathways through PTK7. Furthermore, PTK7 has been identified as a protein with an important role not only in embryogenetic tube formation, but also migration and invasion of endothelial and cancer cells in vitro [10], [11]. While PTK7 expression is upregulated in various cancers including colon, lung, gastric, breast cancer, and acute myeloid leukemia, PTK7 is also considered as a gene involved in the initiation of tumorigenesis [4], [12]–[17]. Therefore, PTK7 plays an important role in the motility and invasivity of cancer cells. However, the biological significance of PTK7 in human BC and lymph node (LN) involvement has not been investigated so far.

In this study we determined the transforming potential of PTK7, and investigated its role in mediating BC cell motility and invasivity. We also analysed mRNA expression of PTK7 in human BC and ipsilateral axillary LN by RT-PCR. PTK7 expression was also compared with some important BC related genes, like HER2, HER3, PAI1, K19, MMP1, and CD44 to understand the role of PTK7 in BC progression and metastasis.

## Materials and Methods

We obtained ethics approval for this project from the ethics committee of the LMU (Ludwig-Maximilians-University) Munich. Patients gave written informed consent for the use of biological materials and relevant clinical data.

### Cell Lines

Cell lines Hs578T and MDA-MB-157 (basal like) were obtained from ECACC; MDA-MB-453,Sk-BR-3, BT-474, T-74D, ZR-75-1 and MDA-MB-175 VII (luminal), BT-20, MDA-MB-468, MDA-MB-231, MDA-MB-435S, and BT-549 (basal like), NIH3T3, HEK293 from ATCC, MDA-MB-361, BT-483, and ZR-75-30 (luminal) from Sugen; MCF7 (luminal), MDA-MB-436 (basal like) and MDA-MB-415 (luminal) from DKFZ. MCF10A (basal like) were a generous gift by B. Gillies [18], SUM-149PT (basal like) by H. Hermeking [19].

All cell lines were authenticated in 2012 using the StemElite ID System (Promega, Madison, WI)

### Antibodies

PTK7-specific antibody was generated by immunization of rabbits with recombinant GST-PTK7 extracellular domain containing amino acid residues 1-703 (PTK7-XCD). For antibody treatment of cells, PTK7-XCD polyclonal antibodies were purified on protein A Sepharose-affinity columns. Anti-αTubulin antibody was obtained from Sigma (St. Louis, MO).

### Immunoblot Analysis

For immunoblotting, cells were washed with ice-cold PBS and then lysed on ice in Triton X-100 lysis buffer (50 mM HEPES, 150 mM NaCl, 10% Glycerol, 1 mM EDTA, 1% Triton X-100, 10 mM Na4P2O7, 10 µg/mlAprotinin, 1 mM phenylmethylsulfonyl fluoride, 10 mM NaF, 1 mM Na3VO4). Clarified whole-cell lysates were separated by SDS/PAGE and transferred to Protran membrane (Whatman). For assessing the expression level of PTK7, the membranes were probed with anti-PTK7-XCD antiserum (1∶20000 dilution) and reprobed with anti-αTubulin for the loading control.

### Generation of Expression Constructs

The 3.2-kbp cDNA sequence coding for PTK7 wild type (PTK7wt) were inserted into the EcoRI/XbaI restriction sites of pcDNA3 (Invitrogen) or the retroviral vector pLXSN. The dominant-negative kinase domain deletion-mutant (PTK7DN) was generated by subcloning the 2.3-kbp EcoRI/XbaI fragment coding for AA1-736 into the same vectors. The extracellular domain-containing construct PTK7XCD was generated by subcloning the 2.1 kbp fragment coding for AA1-703into a GST-expressing pcDNA3 vector.

### Construction of Stable Cell Lines

Hs578T cells were stably infected with PTK7DN or empty vector using supernatants of the amphotropic retrovirus producing cell line PhoenixA which had been transiently transfected with pLXSN constructs. After selection with 1 mg/ml G418 for 14 days cell lines were selected for high expression of PTK7DN, as monitored by Western blot analysis.

HEK293 cells were stably transfected with pcDNA3 constructs containing PTK7XCD using Lipofectamine 2000 (Invitrogen).

### RNA Interference

Transfection of a pool of four 19-nucleotide siRNA duplexes (ON-TARGETplus, Thermo scientific) was carried out using Lipofectamine RNAiMAX (Invitrogen) and OPTI-MEM medium (GIBCO) without FBS. Cells were seeded for Oris cell migration assay 24 hrs and for in-vitro migration assay 48 hrs post-transfection.

### Focus Formation Assay

For Focus Formation Assays NIH3T3 cells were infected with PTK7wt, PTK7DN, empty vector, or v-src as a positive control using supernatants of the ecotropic retrovirus-producing cell line PhoenixE which had been transiently transfected with pLXSN constructs. 2×10^5^ cells were seeded into 6 cm-dishes 4 days after infection and left to grow in DMEM containing 4% FBS for 11 days with changing of media every two to three days. Cells were then stained and fixed with 0.5% Cristal Violet/20% Methanol.

### Soft-Agar Colony Formation Assay

NIH3T3 cells were infected as for Focus Formation Assay. 3.9×10^4^ cells in a top layer of 0.2% Agar Gel (Invitrogen) were seeded onto a bottom layer of 0.7% Agar Gel and left to grow for 16 days. Colony formation was visualised with a Zeiss AxioObserver.A1 microscope.

### Matrigel Assay

For Matrigel outgrowth assays, cells were seeded at 2.5×10^4^ cells per well into 96 well dishes which had previously been coated with 3% Matrigel (BD Biosciences, Bedford, MA). Colony outgrowth was visualized after three days with a Zeiss Axiovert S100 microscope.

### Matrigel Invasion Chamber Assay

Cells were plated at 2×10^4^ cells per well into Matrigel Invasion Chambers (BD Biosciences, Bedford, MA) using DMEM +0.1% FBS +1 µg/ml Mitomycin. The lower compartments contained DMEM +1% FBS +1 µg/ml Mitomycin. After 20 hours incubation, cells were fixed and stained with 20% methanol, 1% crystal violet and cotton swabs were used to remove the cells from the upper side of the membrane. An Axio Observer.A1 microscope (Carl Zeiss, Jena, Germany) was used to record 5 micrographs per well. Photoshop CS5 (Adobe, San Jose, CA) and a proprietary script were used for automated quantification of migration.

### Oris™ Cell Migration Assay

Oris™ Cell Seeding Stoppers (Platypus Technologies, Madison, WI) were inserted into the wells of a 96 well dish to create the migration zone. Cells were then seeded at 9×10^3^ cells per well and incubated for 18 hours to allow cell adherence. Stoppers were then removed and Mitomycin was added to the growth medium at a concentration of 1 µg/ml to inhibit cell proliferation. Cells were permitted to migrate into the wound area for 32 hours and were fixed and stained with 20% methanol, 1% crystal violet. Micrographs of the wound area were recorded with a Zeiss Axio Observer.A1 microscope and the size of the wound area was measured using the MetaVue imaging software (Molecular Devices).

### In vitro Migration Assay

Cells were plated at 2.5×10^4^ cells per well into transwell migration inserts (BD Biosciences, Bedford, MA) using DMEM +0.1% FBS+1 µg/ml Mitomycin. The lower compartments contained DMEM +1% FBS+1 µg/ml Mitomycin. After 4.5 hours incubation, cells were fixed and stained with 20% methanol, 1% crystal violet and cotton swabs were used to remove the cells from the upper side of the membrane. An Axio Observer.A1 microscope (Carl Zeiss, Jena, Germany) was used to record 5 micrographs per well. Photoshop CS5 (Adobe, San Jose, CA) and a proprietary script were used for automated quantification of migration.

### Analysis of Oncomine Data

The Oncomine database tool [20] was used to analyze mRNA expression microarray data from several BC studies (meta-analysis of gene expression of PTK7 as a cancer target). Briefly, PTK7 gene was queried in the database and the results were filtered by selecting BC (Reporter ID: NM_002821). The data from study classes of benign vs. cancer were used for heat-maps. P-values for each group were calculated using student t-test. Standardized normalization techniques and statistical calculations are provided on the Oncomine website and published [21], [22].

### Patients and Tissue Collection

As shown in Table 1, 128 breast cancer patients (median age 59 years, range 27–87 years) with 83 (69%) infiltrated LN, as well as 38 (31%) non-infiltrated LN were included at the Red Cross Women's Hospital Munich in the time period of 2006–2010. Patients with neoadjuvant systemic therapy were excluded. Surgery on breast and axillary was performed stage adapted. BC tissue and LN were collected and stored immediately at −80°C since preparation. To ensure the histological status of harvested LN, they were bisected, with one half submitted for routine histology and the other half taken for examination by RT-PCR. Tumor grade, TNM-classification, and the histopathological tumor subtype were recorded. Hormonal receptor (ER/PR)- and HER2-status was assessed by immunohistochemistry (IHC). Thereby 17 (13%) patients were identified with ER-, PR- and HER2- status, classified as triple negative breast cancer (TNBC). mRNA expression levels of PTK7, BC related genes (HER2, HER3, PAI1, MMP1, K19, CD44), and α-Tubulin as a housekeeping-gene were investigated using RT-PCR.

**Table 1:** Clinico-pathological features of patients.

Variable		Number (%)
Age	≤50 years	42 (33)
	>50 years	86 (67)
Sex	Female	124 (97)
	Male	4 (3)
Menopausal status	Pre	42 (33)
	Post	82 (67)
Histopathological subtype	Invasive ductal	104 (81)
	Invasive lobular	17 (13)
	Other	7 (6)
Tumorstatus	pT1	40 (31)
	pT2	65 (51)
	pT3	12 (9)
	pT4	11 (9)
Lymph node status	pN0	33 (26)
	pN1	47 (37)
	pN2	29 (23)
	pN3	19 (15)
Distant metastasis	M0	120 (94)
	M1	8 (6)
Grading	Grade 1	10 (8)
	Grade 2	75 (59)
	Grade 3	42 (33)
	Unknown	1 (1)
Estrogen receptor status	ER−	32 (25)
	ER+	96 (75)
Progesterone receptor status	PR−	48 (37)
	PR+	80 (63)
Her2/neu status	Her2/neu−	103 (80)
	Her2/neu+	23 (18)
	Unknown	2 (2)

### Total RNA Isolation

Total RNA was isolated using the acid guanidine thiocyanate-phenol-chloroform extraction method [23]. After Na-acetate treatment the total RNA was extracted with phenol-chloroform, precipitated with isopropanol, resuspended with lysis buffer, reprecipitated with isopropanol, washed with 80% ethanol, and finally dissolved in diethyl pyrocarbonate treated H_2_O. The total RNA concentration was determined by absorbance measurement (260/280 nm) and the quality of total RNA was verified by 1% agarose gel electrophoresis. Only samples with no evidence of DNA contamination and RNA degradation were used for cDNA synthesis.

### cDNA-Synthesis

cDNA synthesis was done using 5 µg of total RNA in an oligo(dT) primer mix (MWG Biotech; total volume 10 µL), which was heated at 70°C for 3 min and then cooled on ice. A mastermix containing 1× reverse transcriptase buffer, 1 mmol/L deoxynucleotide triphosphates, 10 mmol/L DTT, 40 unitsRNase inhibitor (40 u/µL; Fermentas), and 50 units avian myeloblastosis virus reverse transcriptase (25 u/µl; Molecular Diagnostics, Roche) was added and subsequently incubated at 42°C for 2 h. A stop reaction was done with 80 µL Tris-EDTA 10/0.1 followed by heating at 72°C for 7 min. The quality of the cDNA was verified using 1.5% agarose gel electrophoresis. Afterwards, the cDNA probe was denatured with 10 µL 1 N NaOH, pH neutralized with 5 µL 2 N HCl and 5 µL 2NTris-HCl (pH 7.5), and purified using the QIAquick PCR Purification kit (Qiagen) according to the manufacturer's instructions.

### RT-PCR

The PCR method was used to determine mRNA expression levels of PTK7, HER2, HER3, MMP1, PAI1,CD44, CK19 and α-Tubulin (housekeeping gene) in fresh-frozen BC and LN samples. The following primer sequences were applied: PTK7-ex 7fwd5′- GGA AGC CAC ACT TCA CCT AGC AG -3′, PTK7-ex11rev 5′-CTG CCA CAG TGA GCT GGA CAT GG -3′, HER2a fwd 5′- CAC ATG ACC CCA GCC CTC TAC AGC -3′, HER2a rev 5′- CAC GGC ACC CCC AAA GGC AAA AAC -3′, HER3 fwd 5′- CTC CGC CCT CAG CCT ACC AGT T-3′, HER3 rev 5′-TGC TCC GGC TTC TAC ACA TTG ACA-3′, MMP1 few 5′- CGA CTC TAG AAA CAC AAG AGC AAG A -3′, MMP1 rev 5′- AAG GTT AGC TTA CTG TCA CAC GCT T -3′, PAI-1 fwd 5′- GCT GAA TTC CTG GAG CTC AG -3′, PAI1 rev 5′- CTG CGC CAC CTG CTG AAA CA -3′, CD44 fwd 5′- GAT CCA CCC CAA TTC CAT CTG TGC -3′, CD44 rev 5′- AAC CGC GAG AAT CAA AGC CAA GGC -3′, K19 fwd 5′- GAG GTG GAT TCC GCT CCG GGC A -3′, K19 rev 5′- ATC TTC CTG TCC CTC GAG CAG -3′, α-TUBfwd 5′- AAG TGA CAA GAC CAT TGG GGG AGG -3′, α-TUB rev 5′- GGG CAT AGT TAT TGG CAG CAT -3′. All primers were synthesized by MWG Biotech. PCR was done using 1 µL cDNA as template in a mastermix containing 1× PCR buffer, 1 mmol/L deoxynucleotide triphosphates, 1 pmol/µL of each specific primer, and 2.5 IU Taq DNA polymerase (Sigma Inc., USA) for each sample and carried out in a thermal cycler (Eppendorf). PCR cycling conditions began with an initial DNA denaturation step at 94°C for 2 min followed by 32 cycles with denaturation at 94°C for 30 s, primer annealing at the appropriate annealing temperature for 30 s, extension at 72°C for 1 min and followed by a final extension step at 72°C for 5 min. The amplified PCR products were analyzed by separation in a 2% agarose gel at 80 V for 30 min. The mRNA expression of the PCR products, with a size of approximately 100 bp- 1 kbp, was evaluated semiquantitatively by using Aida Image Software (Raytest, Germany).

### qRT-PCR for cell lines

Quantitative real time-PCR assays of breast cancer cell lines were performed using the Applied Biosystems SteponePlus® detection system. The PTK7 specific primer sequences were fwd 5′- CAG TTC CTG AGG ATT TCC AAG AG -3 and rev 5′- TGC ATA GGG CCA CCT TC -3′. Amplification of α-Tubulin as an endogenous reference was performed to standardize the amount of sample mRNA. α-Tubulin primers (fwd 5′- CCG GGC AGT GTT TGT AGA C -3, rev 5′- GTC CCA GTC CGA ACT TC -3′) and probes were purchased from Thermo Fisher Scientific. The PCR was carried out with Solaris qPCR Master Mix using 1 µL of cDNA, diluted 1∶20 in H_2_O and 1 µL 20x Solaris primer set in a final volume of 10 µL. The reactions were incubated in a 96-well optical plate at 95°C for 15 min, followed by 40 cycles of 95°C for 15 s and 60° for 1 min. Experiments were performed in duplicate for each sample and Ct data of each sample was determined using default threshold settings. Relative quantification of mRNA expression was calculated with the 2^−ΔΔCt^ method. Results were reported as average expression +- SEM.

### Statistical analysis

Statistical analysis was done using R 2.13.2 (R Foundation for Statistical Computing, Vienna, Austria).

Considering the PTK7 expression in BC cell lines, groups were compared using exact Mann-Whitney U (MW) tests. Data are presented as median (range).

For clinicopathological evaluation of PTK7 expression the following clinically relevant variables were considered for statistical analysis: Tumor grade, TNM-classification, steroidhormonal receptor-, and HER2-status. To test for differences in PTK7 expression in these variables one-way ANOVA was used for the comparison of more than two groups, otherwise two sample t- tests were used. Data are presented as mean ± standard deviation.

Pearson correlation coefficients were used to evaluate the level of correlation between HER2, HER3, PAI1, MMP1, CD44 and PTK7 expressions.

For the comparison of not infiltrated LN and LN metastasis in respect of gene expression the Mann-Whitney U test was used.

Survival analysis end points were overall survival (OS) and disease free survival (DFS). DFS and OS were determined in months from the date of surgery. DFS end points corresponded with the detection of a recurrent tumor or distant metastasis or death. Overall survival was measured from the time of surgery until death or last follow-up. Survival data are presented using the Kaplan-Meier life-table method; differences between groups were tested by long-rank tests. Hazard ratios were calculated using the Cox proportional hazards model. To illustrate the effect of PTK7 expression on DFS the PTK7 expression was divided in low and high according to the median of PTK7 expression. A significance level of 5% was used. All tests were performed two sided.

## Results

### Transforming potential of PTK7

The ability of cells to grow independently of their attachment to tissue culture surfaces and to overcome contact inhibition is a strong indicator of transforming potential.

**Figure 1:**
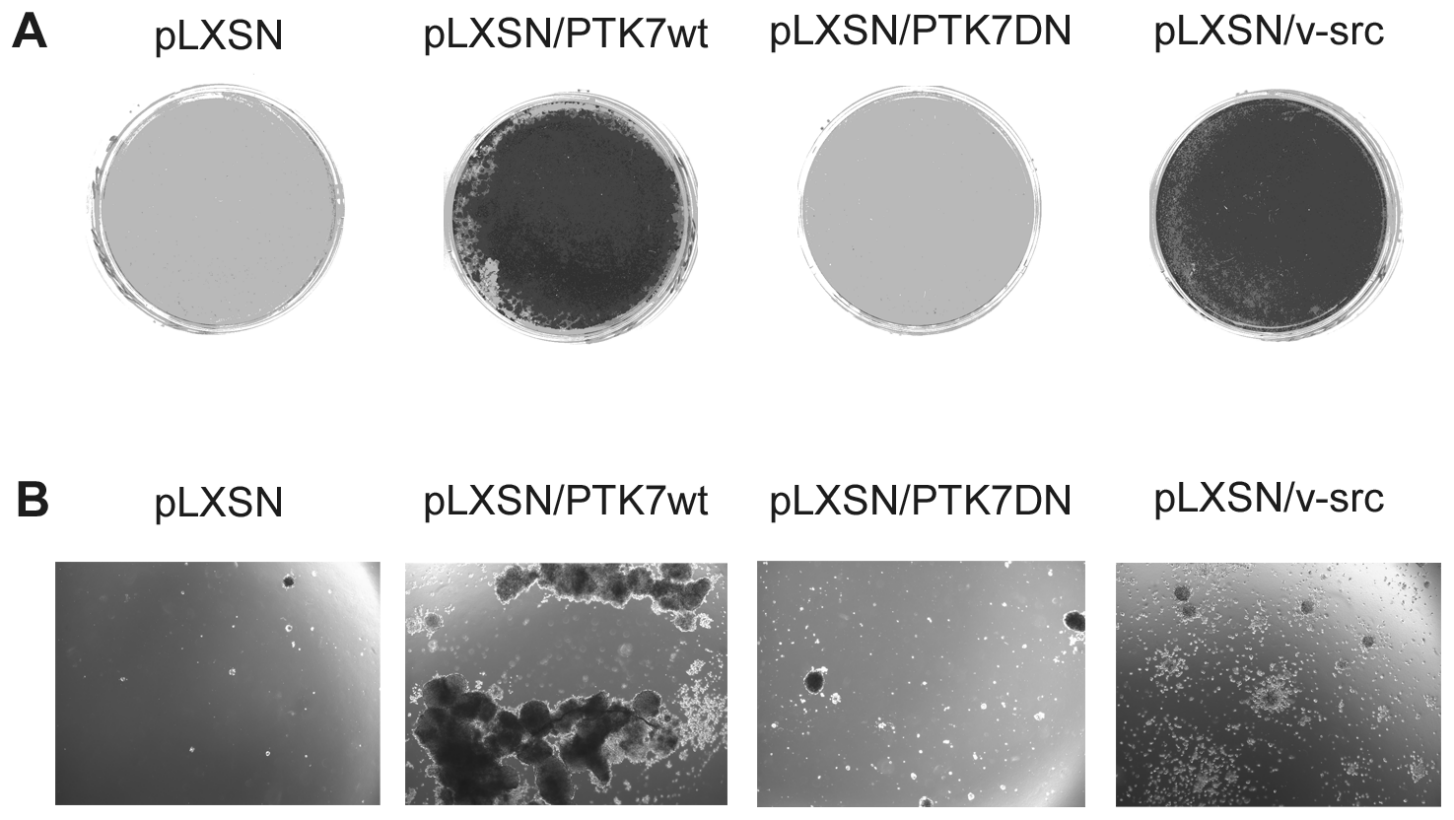
PTK7 is able to confer oncogenic potential to NIH3T3 cells. NIH3T3 cells were infected with PTK7wt, PTK7DN, empty vector or v-src as a positive control and then seeded for Focus Formation Assay (A) or Colony Formation Assay in Soft Agar (B).

After transient infection of NIH3T3 cells with pLXSN/PTK7wt, pLXSN/PTK7DN, or pLXSN/v-src (positive control) focus formation and soft agar colony formation assays were performed in order to determine the transforming potential of PTK7. Focus formation was detectable in the case of pLXSN/PTK7wt and pLXSN/v-src infection, but not after infection with pLXSN/PTK7DN. Similar results were observed in soft agar assays with a high number of colonies in pLXSN/PTK7wt infected NIH3T3 cells in contrast to pLXSN/PTK7DN (lower colony number) and mock infected cells (no colonies) (Fig.1), indicating that PTK7 has strong transforming potential.

### Expression of PTK7 in Breast Cancer Cell Lines

**Figure 2:**
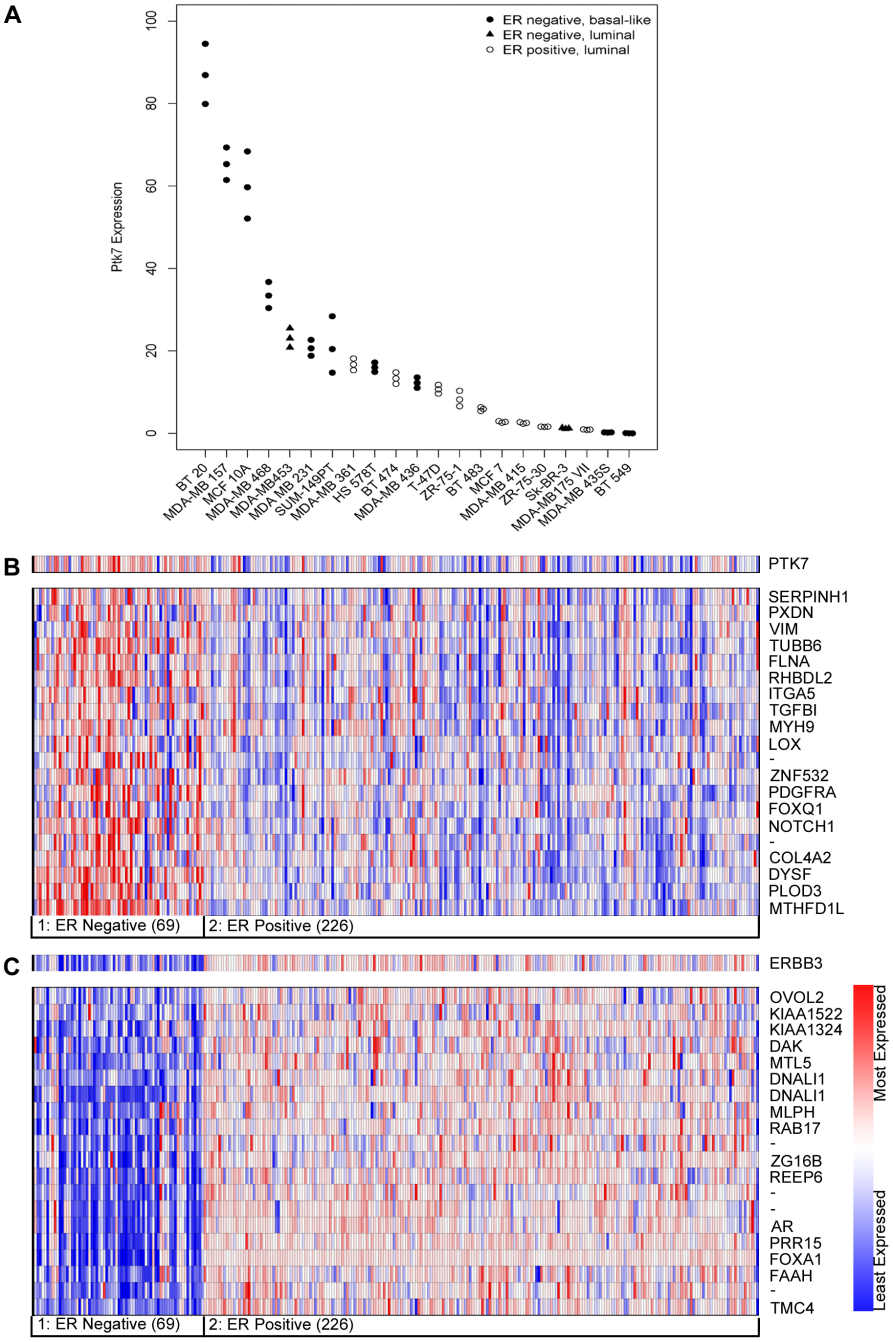
Analysis of PTK7 Expression in BC cell lines and primary tumors. (A) Classification of BC cell lines by PTK7 expression: Cell lines Hs 578T, MDA-MB-157, BT-20, MDA-MB-468, MDA-MB-231, MDA-MB-435S, MDA-MB-436, BT-549, MCF10A1, SUM-149PT classified as basal like, MDA-MB-453, Sk-BR-3, BT-474, T-74D, ZR-75-1, MDA-MB-175VII, MDA-MB 361, BT-483, ZR-75-30, MCF7, and MDA-MB415 classified as luminal cell lines were analysed by RT-PCR. Higher PTK7 expression in BC cell lines which lack expression of ER and are grouped as basal-like. (B) Heat map of genes co-expressed with PTK7 in primary breast carcinomas (van de Vijver, Oncomine), which were grouped by ER status. The colors relate to expression units which are z-normalized to depict relative values within rows. (C) Heat map of genes co-expressed with oncogene HER3 in primary breast carcinomas (van de Vijver, Oncomine).

To identify the role of PTK7 in BC, we determined mRNA expression levels in 1 non tumorigenic mammary epithelial and 20 BC cell lines by qRT-PCR. We grouped them in estrogene receptor (ER) positive (luminal) and ER negative (luminal, basal-like) and examined PTK7 expression. PTK7 expression was increased in ER negative BC cell lines (n = 12) ((median: 20.7 (range: 0.0 to 86.9)) compared to ER positive cell lines (n = 9) ((median: 5.9 (range: 0.9 to 16.7)). This high numerical difference was not statistically significant (p = 0.082) which can be due to small sample sizes. PTK7 overexpression was detected in basal like (TNBC) BC compared to luminal BC cell lines (p = 0.040, Figure 2A). Immunoblots of the used cell lines confirm this expression pattern (Figure S1).

### Oncomine Data

We have conducted a study where Oncomine microarray data [24] were analyzed to evaluate PTK7 gene expression in BC tumors. The PTK7 gene is differentially expressed in BC patients based on their ER status. High expression was observed in ER negative tumors compared to ER positive (Figure 2B) especially, if simultaneously HER3-expression was low (Figure 2C). Furthermore we identified a cluster of genes significantly co-expressed with PTK7 which comprises Vimentin (Figure 2B), DDR2, EphA2, PDGFR-alpha, and metalloproteinases MMP2, MMP3, MMP-14, TIMP2, and ADAM12 (Data not shown). These genes are well known as associated with aggressive behaviour of primary BC. Since signalling of PTK7 is connected to the Wnt-pathway, some genes were clustered like Wnt2, Jag1, and Frizzled (Data not shown). Interestingly, GAS6, the ligand for RTK AXL, is also co-expressed with PTK7 (Data not shown) [25]–[31]. It is known that the AXL-GAS6 axis of signalling is connected to TNBC and poor prognosis in BC progression. To summarize, PTK7 overexpression was detected in TNBC patients and correlated with poor prognosis in this cohort.

### PTK7 expression correlates with motility and invasivity of breast cancer cells

**Figure 3:**
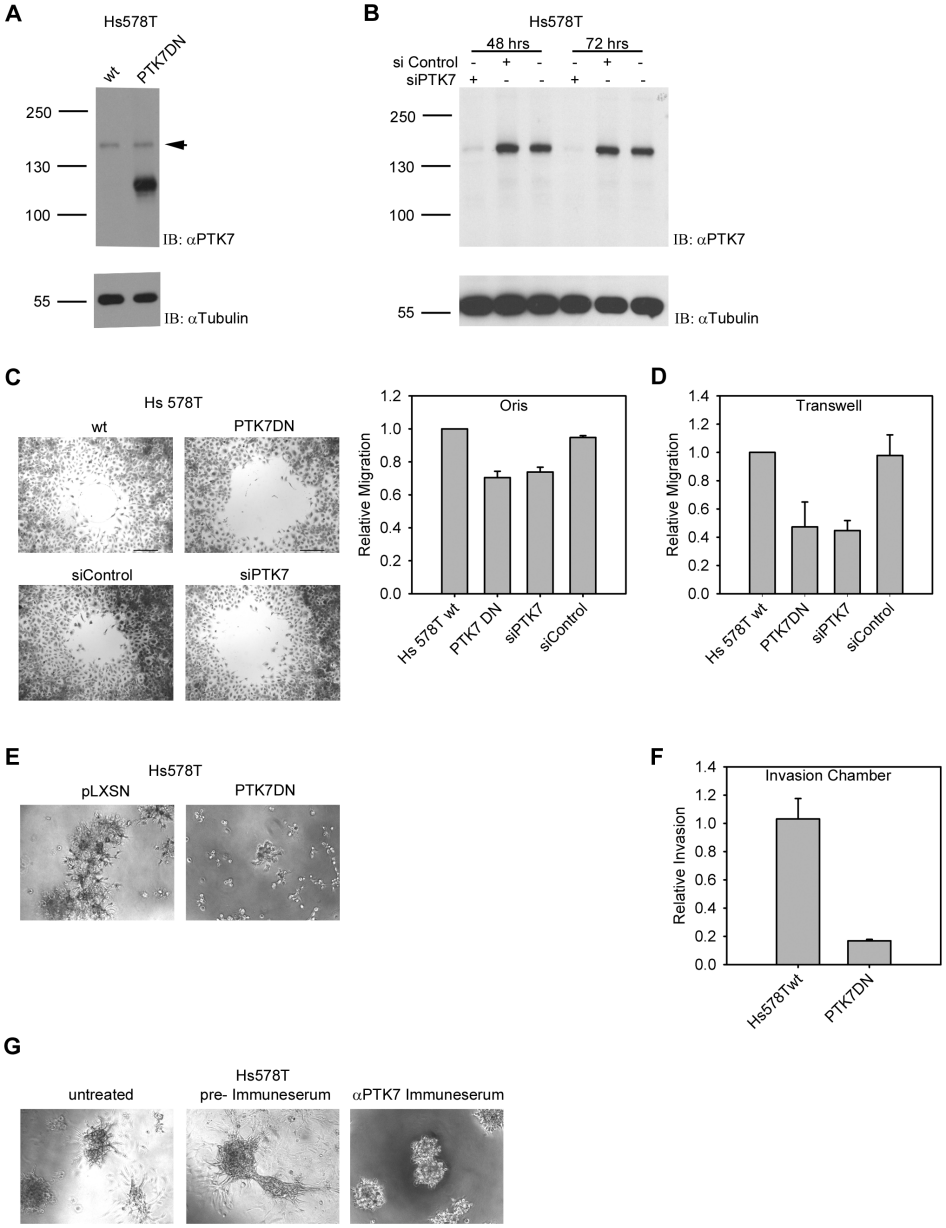
Expression of full length PTK7 correlates with motility and invasivity of breast cancer cells. (A) Expression of full length and dominant negative PTK7 protein in wild type or PTK7DN overexpressing Hs578T cells which were subsequently used in cellular assays. Whole cell lysates were used to detect the protein level of PTK7 by immunoblot analysis (top) with α-Tubulin as loading control (bottom). (B) Expression of PTK7 protein 48 and 72 hrs after siRNA transfection. Whole cell lysates were used to detect the protein level of PTK7 by immunoblot analysis (top) with α-Tubulin as loading control (bottom). (C) The motility of cells was analysed by Oris™ cell migration assay. Cells were allowed to migrate into the wound area for 32 hrs. Cell migration was visualized at 4× magnification, representative micrographs are shown (scale bar  = 500 µm). Error bars indicate SEM (n = 3). (D) In vitro migration assay. Cells were allowed to migrate through transwell inserts for 4.5 hours. Error bars indicate SEM (n = 3). (E) The invasivity of cells was analysed by Matrigel outgrowth assay. Cells were seeded on the surface of Matrigel. Colony outgrowth was visualized at 10× magnification. (F) Matrigel Invasion Chamber Assay. Cells were allowed to invade through the Matrigel matrix and membrane pores for 20 hours. Error bars indicate SEM. (G) Effect of an anti-PTK7 polyclonal antibody on the invasivity of Hs578T cells in Matrigel outgrowth assay. Cells were seeded on the surface of Matrigel and treated with anti-PTK7 antibody or control antibody for 3 days. Colony outgrowth was visualized at 10× magnification.

In order to study the role of PTK7 in motility and invasivity of TNBC cells, dominant-negative inhibition of PTK7 through overexpression of a kinase domain deletion mutant (PTK7DN) in the highly invasive, PTK7-expressing BC cell line Hs578T was applied (Figure 3A). In an alternative approach, we examined the effects of siRNA mediated PTK7 knockdown on the motility of Hs578T cells. Cell motility was examined by the Oris™ cell migration assay. Hs578T control cells migrated into the wound at a higher rate than both the PTK7DN-overexpressing or siRNA-transfected Hs578T cells (Figure 3C). This observation was confirmed by decreased migration toward FBS as a chemoattractant in transwell migration assays (Figure 3D).

Invasive behaviour of cancer cells is reflected in cell culture by their ability to grow into Matrigel. When cell invasivity was examined by the Matrigel outgrowth assay, as shown in Figure 3E, Hs578T mock transfected control cells formed stellate colonies with filopodial structures invading the surrounding matrix; in contrast, the corresponding PTK7DN-overexpressing cells were unable to form such outgrowth structures. This observation was substantiated by the decreased abiltity of PTK7DN-overexpressing cells to detach themselves from and invade through the Matrigel matrix and membrane pores of Matrigel Invasion Chambers toward FBS as a chemoattractant (Figure 3F). Therefore, we concluded that overexpression of PTK7DN converted the highly invasive phenotype of breast cancer cells to a weakly or noninvasive phenotype.

### Suppression of cancer cell invasivity by an anti-PTK7 polyclonal antibody

To evaluate the efficacy in vitro of a more therapy-like intervention strategy, we generated a polyclonal antiserum against the extracellular domain of PTK7 and studied its effect on the invasivity of Hs578T cells in Matrigel assay. PTK7 antibody treated cells grew only as spherical clusters on the surface without potential of penetration into the Matrigel in contrast to cells which were treated by control antibody (Figure 3G).

### PTK7 expression and clinico-pathological parameters in primary breast cancer tissue

We investigated whether PTK7 overexpression in ER-negative BC cell lines also reflects the expression status in ER-negative human breast tumors and LN metastasis. Therefore, we evaluated the hormonal receptor (HR)- and HER2 receptor-status in relation to PTK7 expression. Whereas there was no statistically significant difference in BC (mean difference: −0.18, 95% CI: −0.89 to 0.52, p = 0.602), PTK7 expression in LN metastasis was strongly linked to ER- status (mean difference: 1.26, 95% CI: 0.46 to 2.06, p = 0.003), PR- status (mean difference: 0.87, 95% CI: 0.09 to 1.66, p = 0.030) and HER2-status (mean difference: -1.5, 95% CI: −2.39 to −0.65, p = 0.001). In grouped analysis for PTK7 expression in ER/PR/HER2-negative (TNBC) vs. ER/PR/Her2-positive BC there was a tendency for higher expression in TNBC, but was not statistically significant due to a small sample size of TNBC (n = 14) and ER/PR/Her2-positive (n = 6) cohort (mean difference −1.55, 95% CI: −3.36 to 0.25, p = 0.083, t-test). In univariat analysis significant differences in PTK7 expression for tumor size (ANOVA, p = 0.033) in BC and nodal status (ANOVA, p = 0.007) in LN (higher expression level with number of LN metastasis) was seen (Table 2).

**Table 2:** Association of PTK7 expression and patient's clinico-pathological variables in primary tumors and lymph nodes of 128 breast cancer patients.

		PTK7 Expression					
		BC			LN		
Clinicopathological parameters		N	mean ± SD	p-value	N	mean ± SD	p-value
Tumorsize	pT1	35	3.5±1.8	0.033	20	2.5±1.7	0.828
	pT2	58	3.0±1.5		44	2.3±1.6	
	pT3	9	3.2±1.6		10	3.0±2.3	
	pT4	10	2.2±1.7		9	2.4±2.2	
Nodal status	pN0	28	2.7±1.8	0.385	2		0.007
	pN1	44	3.2±1.6		40	2.0±1.8	
	pN2	25	3.3±1.5		23	2.4±1.7	
	pN3	15	3.1±1.9		18	3.5±1.6	
Grading	G1	9	3.3±0.6	0.661	5	2.5±1.2	0.094
	G2	64	3.1±1.7		50	2.1±1.7	
	G3	38	3.1±1.7		27	3.0±2.0	
ER status	ER−	28	3.0±1.6	0.602	22	3.4±1.5	0.003
	ER+	84	3.1±1.7		61	2.1±1.7	
PR status	PR−	43	3.1±1.7	0.907	32	3.0±1.8	0.030
	PR+	69	3.1±1.7		51	2.1±1.7	
Her2/neu	Her2/neu−	88	3.0±1.6	0.162	64	2.1±1.7	0.001
	Her2/neu+	22	3.5±1.7		18	3.6±1.6	
Grouped receptor status	ER, PR, Her2/neu−	13	2.7±1.5	0.083	9	3.2±1.9	0.884
	ER, PR, Her2/neu+	6	4.2±1.5		5	3.4±1.3	

In order to validate these qPCR data, PTK7 immunohistochemistry was performed with 35 BC and 35 LN tissue samples of breast cancer patients. The results are shown as supporting information.

### Co-expression of Breast Cancer Related Genes and PTK7

**Figure 4:**
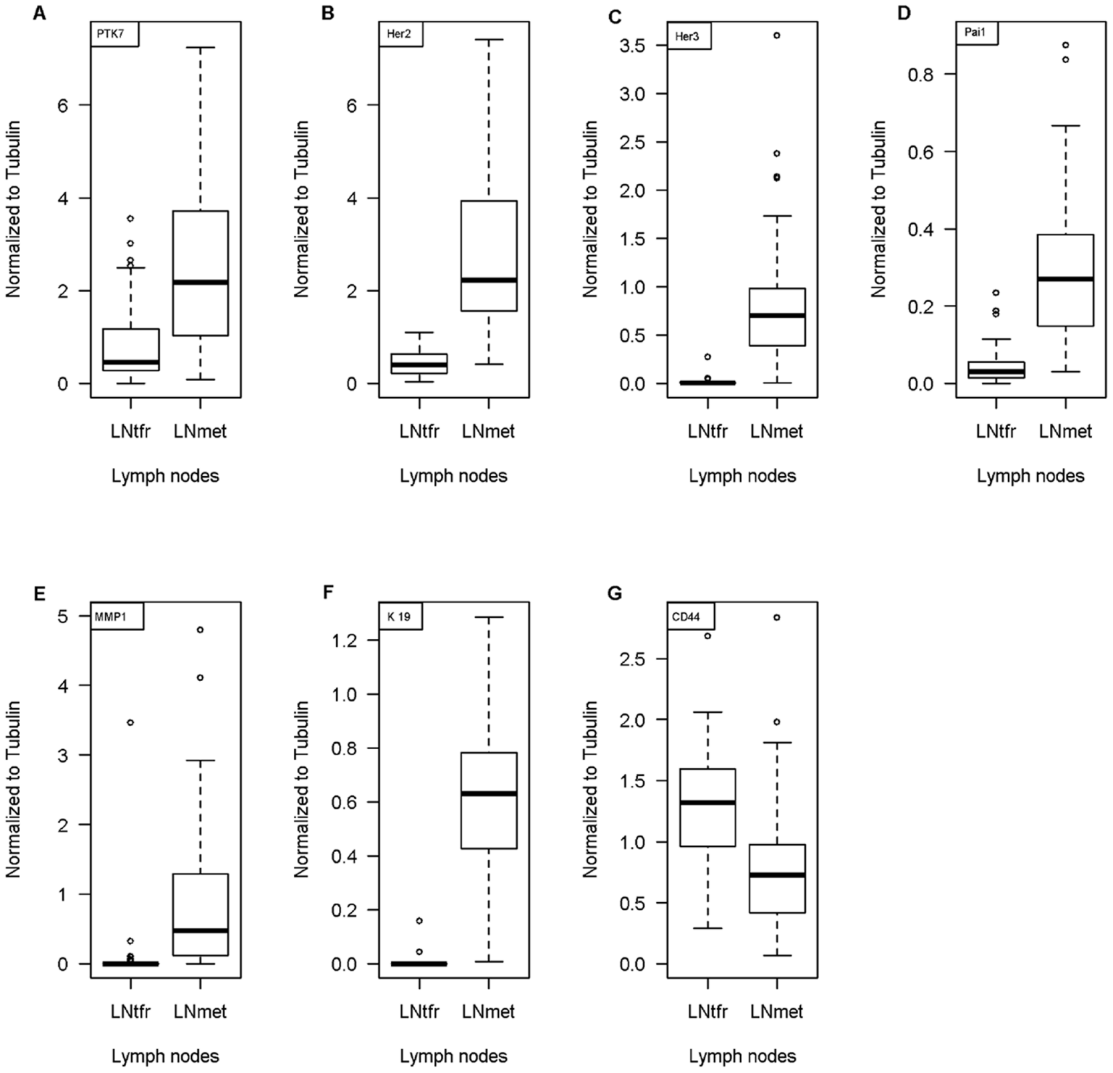
Co-expression of Breast Cancer Related Genes and PTK7. PTK7 and BC-related genes (Her2, Her3, Pai1, MMP1, CK19 and CD44) expression was assessed by RT-PCR and compared in LN metastasis (LNmet) and tumorfree LN (LNtfr) of 128 patients. All genes (PTK7, Her2, Her3, Pai1, MMP1, CK19) showed an overexpression in LN metastasis compared to tumorfree LN.

To assess the tumorigenic potential of PTK7 in BC and LN we compared the PTK7 expression level and co-expression with other breast cancer related genes (HER2, HER3, PAI1, MMP1, CK19, CD44). In primary BC we found significant correlation between PTK7 and PAI1 (r = 0.494, p<0.001), MMP1 (r = 0.286, p  =  0.002) and CD 44 (r = 0.247, p = 0.014). For LN metastasis PTK7 co-expression was seen with HER2 (r = 0.448, p<0.001), PAI1 (r = 0.549, p<0.001) and MMP1 (r = 0.433, p<0.001) (Table 3). When gene expression levels of non-involved LN and LN metastasis were examined, we saw significantly higher expression in LN metastasis for all genes except CD44 (PTK7, HER2, HER3, PAI1, MMP1, CK19) (Fig. 4A–G). Taken together, PTK7 seems to be linked to tumor progression and metastasis like the other genes.

**Table 3:** Co-expression of BC related genes and PTK7 expression in BC and LN metastases as measured by RT-PCR.

PTK7 co-expression of	BC (n = 114)		LN (n = 83)	
Genes	Pearson's r	p-value	Pearson's r	p-value
Her2/neu	0.171	0.077	0.448	<0.001
Her3	0.013	0.897	−0.007	0.953
Pai1	0.494	<0.001	0.549	<0.001
MMP1	0.286	0.002	0.433	<0.001
CK19	0.139	0.144	0.179	0.116
CD44	0.247	0.014	0.184	0.124

### Prognostic role of PTK7 expression

**Figure 5:**
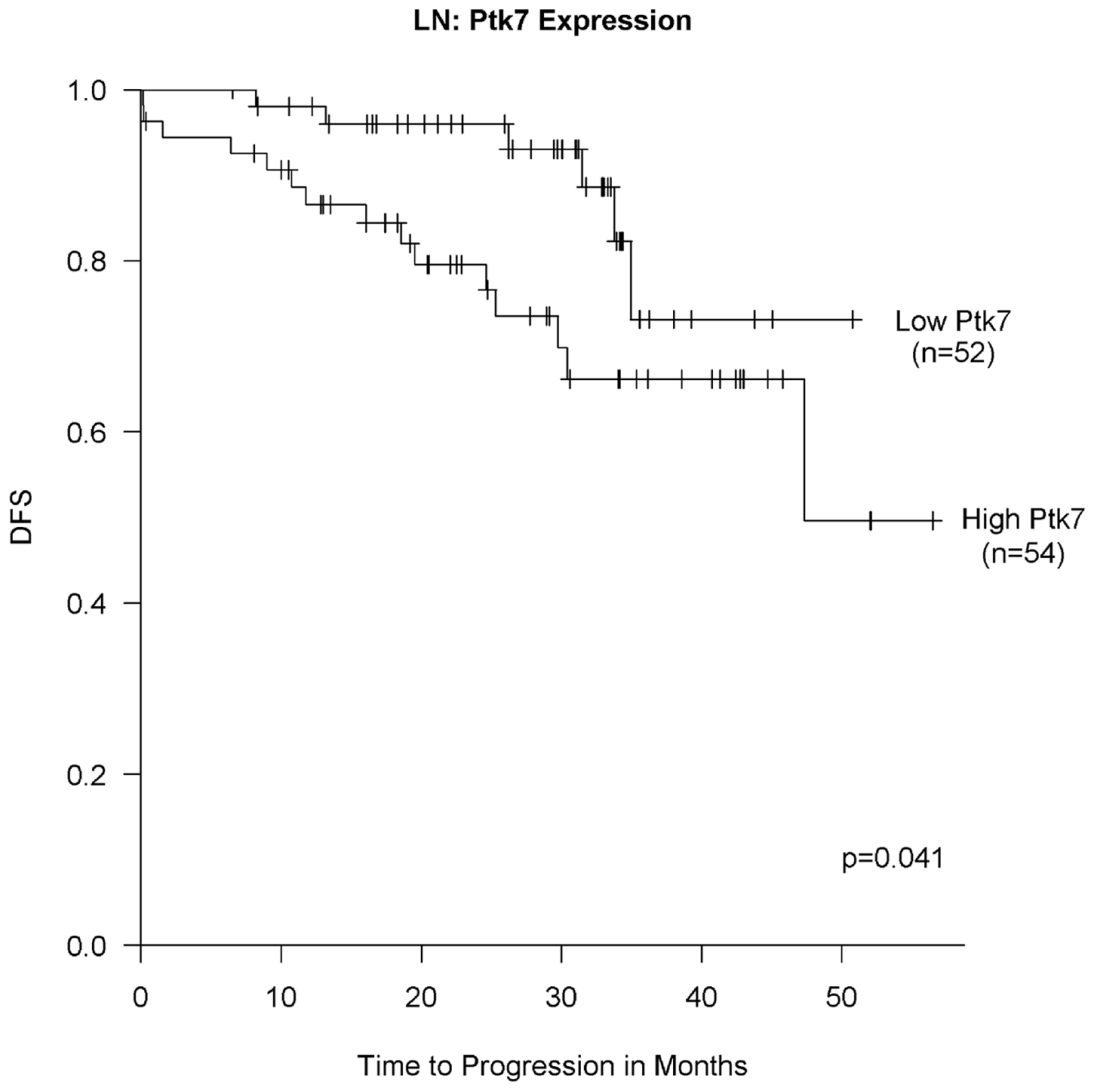
PTK7 serves as a prognostic marker in lymph nodes involvement. Kaplan-Meier curve showing disease-free survival (DFS) in breast cancer patients with lymph node metastasis classified into PTK7 low and high groups according to RT-PCR expression. PTK7 expression is associated with DFS among breast cancer patients with lymph node metastasis expressing high PTK7. P-values were calculated by the log-rank test.

In order to determine the prognostic impact of PTK7 we correlated expression levels with patient survival. According to our hypothesis we further grouped patients in TNBC and non-TNBC cohorts. 118 breast cancer patients were followed-up for a median period of 29 months (9 patients died and 21 relapsed). For OS no correlation was detected with PTK7 expression most likely due to short follow-up time. Patients with higher PTK7 expression in LN metastasis had a significantly shorter DFS with a HR of 1.25 (95% CI: 1.04–1.50, p = 0.016) for every increase of one unit in the PTK7 expression. The estimated 24-months rates for DFS were 96% (95% CI: (0.91–1.00) in the low expression group, as compared with 80% (95% CI: (0.69–0.92); p = 0.041) in the high (Figure 5). For BC and also for group analysis (TNBC vs non-TNBC) no correlation between PTK7 expression and DFS was detected. However, this may be due to limited sample size of TNBC.

## Discussion

In our study we identify PTK7 (originally CCK4) as being differentially expressed together with certain other genes in TNBC compared to ER-positive and normal breast cell lines.

The pseudokinase PTK7 was originally identified as a protein overexpressed in several cancer cell lines, including melanoma [32] and colon [4].

There have been several reports about the upregulation of PTK7 expression in different types of cancer such as pulmonary adenocarcinoma [12], gastric[13] and breast carcinoma [17] but none about its oncogenic potential. In this work we could show that PTK7, like most RTKs including HER3, is indeed able to transform NIH3T3 cells, as shown in Focus Formation and Soft Agar Colony Formation Assays (Figure 1).

Furthermore we could identify PTK7 as a potential mediator of motility and invasivity of BC cells. We found that the expression of PTK7 was correlated with the invasive properties of BC. Inhibition of endogenous PTK7 signaling in highly invasive BC cells with TNBC characteristics by a dominant negative mutant or siRNA silencing results in loss of the capacity to invade the surrounding matrix and to migrate into the wound area. The dominant negative mutant might function here as a competitor of endogenous PTK7 for a yet to be identified ligand or other binding partner, as has been described for a soluble form of PTK7 [11].

Antibodies, by virtue of the fact that they are highly specific, represent an ideal approach for selectively interfering with a specific target molecule. In this study we performed experimental therapy against BC in vitro by using a polyclonal PTK7 antibody, and showed that the antibody successfully inhibited the invasivity of Hs578T breast cancer cells, which proved that developing a therapeutic monoclonal anti-PTK7 extracellular domain antibody is a promising strategy for the treatment of invasive BC.

Treatment decisions for BC are mainly based on clinicopathological variables that are prognostic, such as tumor size, presence of lymph node metastasis, grading, and three predictive markers (ER, PR and HER2). However, current markers do not allow accurate prediction of the likelihood of recurrence, and improvements are needed to clearly identify which women are at sufficiently low risk to be able to safely avoid the use of chemotherapy and its accompanying adverse effects. Present first generation prognostic signatures are clinically useful only in patients with ER positive disease, but not for ER negative patients. The identification of receptor tyrosine kinases as promising targets for directed cancer therapy approaches has further increased the scientific interest in this important protein family. For example, the humanized monoclonal antibody Trastuzumab (Herceptin) has been developed and shows significant clinical activity in HER2 positive BC. Today Herceptin is the therapeutic principle of individualized BC treatment.

As shown above, PTK7 has transforming potential and could therefore be a good candidate for BC treatment and inhibition of BC development. Furthermore, we evaluated the relationships between PTK7 expression and clinical features in BC patients. These results are the first to show that PTK7 overexpression is a novel marker for LN involvement and has additional prognostic impact for LN positive BC patients. PTK7 overexpression in LN metastasis was significantly associated with shorter DFS (Figure 5). Based on these results, we propose that PTK7 could be a novel prognostic marker for BC patient survival.

Even if PTK7 is upregulated in various cancers including lung cancer [12], gastric cancer [33], and acute myolid leukaemia (AML) [15] its function in tumorigenesis and metastasis remains controversial. Endoh et al. [12] calculated a prognostic significant risk index defined as a linear combination of 8 gene expression values weighted by their estimated regression coefficients in pulmonary adenocarcinoma. Interestingly, for PTK7 the coefficient was negative suggesting that high expression of the PTK7 gene was associated with good prognosis (PTK7 HR, 0.50, 95%CI 0.244 to 1.024, p = 0.0582). Similar findings were reported by Lin et al [33] for gastric cancer. PTK7 expression assessed by IHC was an independent prognostic factor for favorable OS (P = 0.028) and DFS (P = 0.012). On the other hand, PTK7-positive AML patients were more resistant to anthracycline-based therapy with a significantly reduced relapse-free survival (RFS) [15]. PTK7 expression is also reported to be associated with significantly worse 3-year DRFS outcome in liposarcoma patients [3]. This may be quite reasonable considering cancer is a complex multigene disease. In our cohort we were not able to see a correlation between PTK7 expression in BC and concurrent LN metastasis or unfavorable survival. But this is not strictly contradictory to published clinical data. In PTK7-positive AML the frequency of extramedullary disease at diagnosis was lower [15] although RFS was reduced. Remarkably, in our study higher PTK7 expression in LN metastasis was not only significantly associated with a higher number of tumor-involved LN, but also with a significantly shorter DFS (96% (95%low vs 80% high after 24 months p = 0.016). Furthermore, PTK7 is thus suitable for discrimination of metastatic LN, PTK7 expression levels were significantly higher in involved LN compared with non-involved LN. Thus, in addition to previously published genes like CK19 and MMP1, PTK7 is a dicriminator for LN metastasis.

In basal like BC cell lines we detected high PTK7 expression levels (Figure 2A). This trend was also seen in the TNBC cell lines compared to “triple-positives”, but this was not statistically significant. Oncomine data show that PTK7 is over-expressed in TNBC and cell lines, moreover, no correlation with LN involvements has been shown. Our data for the first time identified an association of PTK7 with LN infiltration.

Our results highly support the fundamental function of PTK7 in BC. PTK7 overexpression in LN metastasis was associated with a higher amount of involved LN and significantly inferior DFS in BC patients. We were not able to prove our hypothesis generated by Oncomine analysis that PTK7 expression is prognostic in TNBC patients, but in our opinion this was due to small sample size. If so, PTK7 could offer a target in this poor prognostic cohort, where an effective treatment is urgently needed.

## Supporting Information

Figure S1 **Immunoblot Analysis of PTK7 Expression in BC cell lines.** (B) Whole cell lysates were used to detect the protein level of PTK7 in 21 BC cell lines. (A) Band intensity was determined using the AIDA Advanced Image Data Analyzer Software (Raytest, Straubenhardt, Germany).
(TIF)

Figure S2 **Representative cases demonstrating the relation of PTK7 expression in breast cancer by immunohistochemical staining and rtPCR value.** (A) Weak (+) IHC scoring and low PTK7 rt-PCR (0.356) in invasiv-ductal non-TNBC (upper panel: magnification 200× scale bar: 100 µm; lower panel: magnification 400×, scale bar: 50 µm), (B) Strong (+++) IHC scoring and high PTK7 rtPCR (2.496) in invasiv-ductal TNBC (upper panel:magnification 200×, lower panel: magnification 400×), (C) Weak (+) IHC scoring in normal breast tissue (upper panel: magnification 200×, lower panel: magnification 400×).
(TIF)

Table S1 **Association of PTK7 expression and patient's clinico-pathological variables in primary tumors (BC) and lymph nodes (LN) by immunohistochemistry (IHC).**
(DOCX)

Text S1 **Immunohistochemistry.**
(DOCX)
