# Supplementary figures and images for: PTK 7 Is a Transforming Gene and Prognostic Marker for Breast Cancer and Nodal Metastasis Involvement
Source: PLoS One. 2014 Jan 7;9(1):e84472. doi: 10.1371/journal.pone.0084472 (PMC3883666; doi:10.1371/journal.pone.0084472)

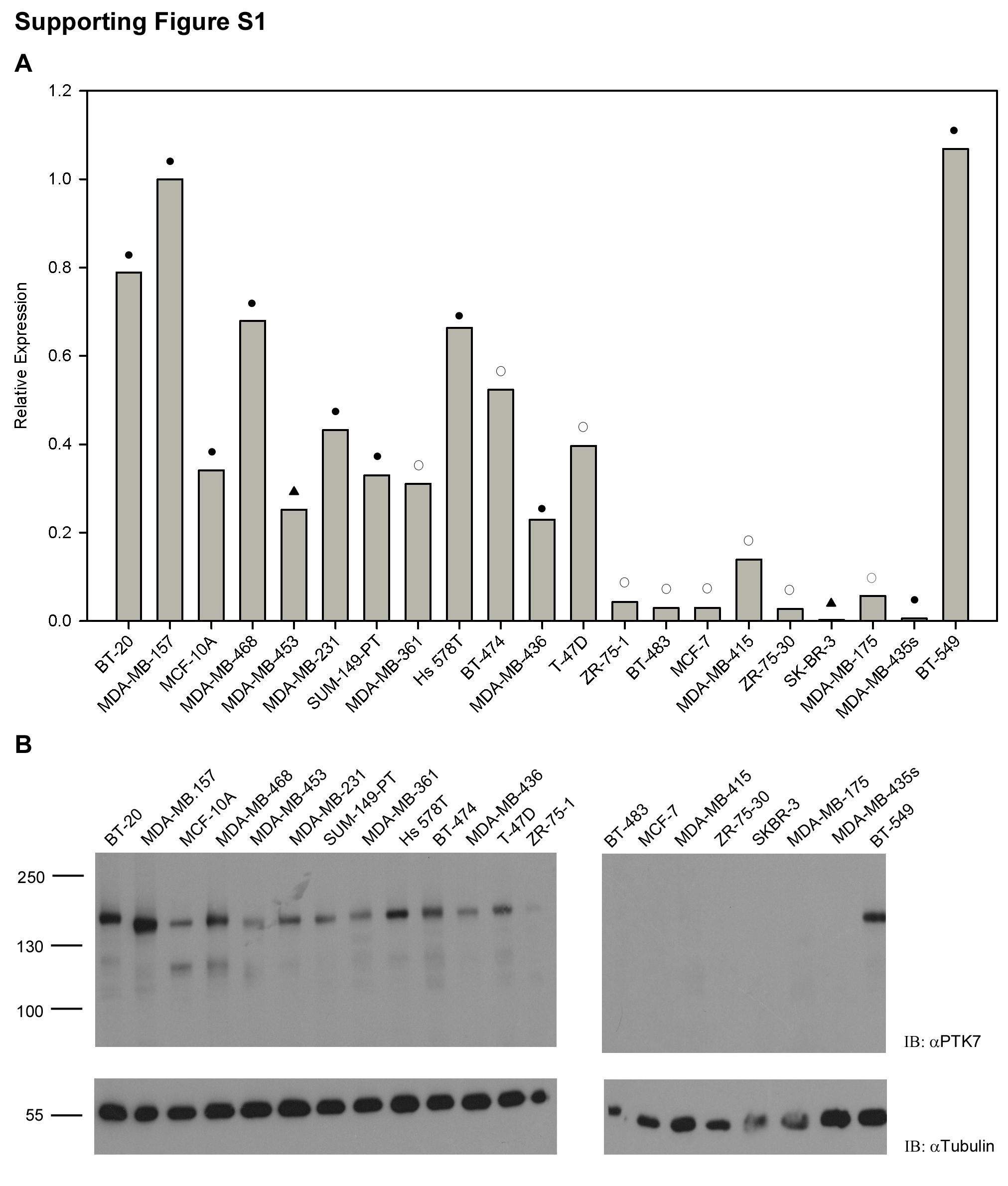

Supplement: Figure S1 — Immunoblot Analysis of PTK7 Expression in BC cell lines. (B) Whole cell lysates were used to detect the protein level of PTK7 in 21 BC cell lines. (A) Band intensity was determined using the AIDA Advanced Image Data Analyzer Software (Raytest, Straubenhardt, Germany). (TIF) [file pone.0084472.s001.tif]

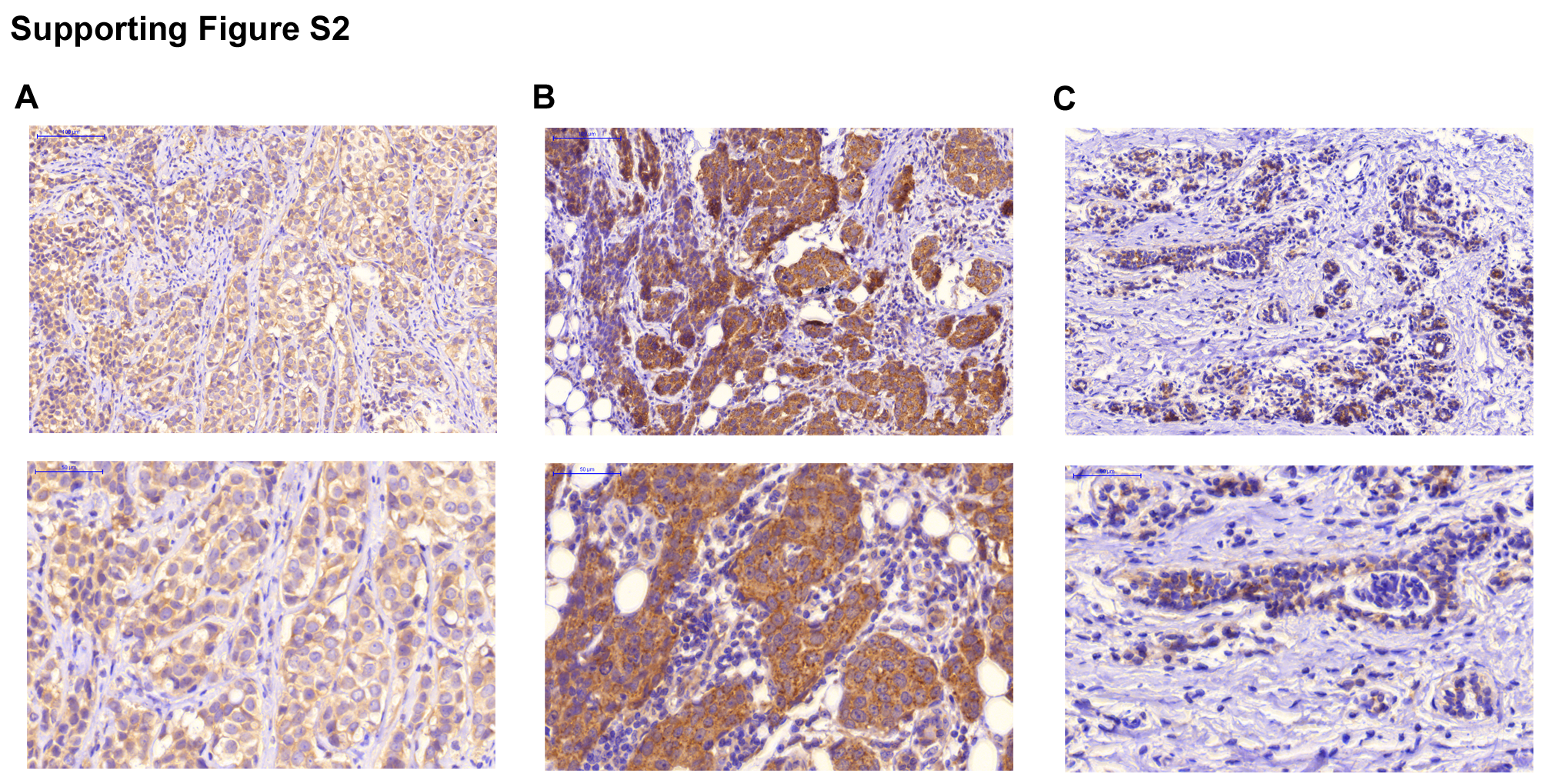

Supplement: Figure S2 — Representative cases demonstrating the relation of PTK7 expression in breast cancer by immunohistochemical staining and rtPCR value. (A) Weak (+) IHC scoring and low PTK7 rt-PCR (0.356) in invasiv-ductal non-TNBC (upper panel: magnification 200× scale bar: 100 µm; lower panel: magnification 400×, scale bar: 50 µm), (B) Strong (+++) IHC scoring and high PTK7 rtPCR (2.496) in invasiv-ductal TNBC (upper panel:magnification 200×, lower panel: magnification 400×), (C) Weak (+) IHC scoring in normal breast tissue (upper panel: magnification 200×, lower panel: magnification 400×). (TIF) [file pone.0084472.s002.tif]
