# Supplementary material for: PTK 7 Is a Transforming Gene and Prognostic Marker for Breast Cancer and Nodal Metastasis Involvement
Source: PLoS One. 2014 Jan 7;9(1):e84472. doi: 10.1371/journal.pone.0084472 (PMC3883666; doi:10.1371/journal.pone.0084472)
Supplement: Table S1 — Association of PTK7 expression and patient's clinico-pathological variables in primary tumors (BC) and lymph nodes (LN) by immunohistochemistry (IHC). (DOCX) [file pone.0084472.s003.docx]

|  |  |  | Positive PTK7 expression | |
| --- | --- | --- | --- | --- |
| **Variable** |  | **Number (%)** | **BC p-value** | **LN p-value** |
| Age | ≤ 50 years | 11 (31) |  |  |
|  | > 50 years | 24 (69) |  |  |
| Sex | Female | 33 (94) |  |  |
|  | Male | 2 (6) |  |  |
| Menopausal status | Pre | 14 (40) | 0.171 | **0.001*** |
|  | Post | 19 (60) |  |  |
| Histopathological subtype | Invasive ductal | 28 (80) | 0.259 | 0.791 |
|  | Invasive lobular | 6 (14) |  |  |
|  | Other | 2 (6) |  |  |
| Lymph node | tumorfree | 10 (29) |  |  |
|  | metastasis | 25 (71) |  |  |
| Tumorstatus | pT1 | 10 (28) | 0.182 | 0.233 |
|  | pT2 | 15 (43) |  |  |
|  | pT3 | 3 (9) |  |  |
|  | pT4 | 7 (20) |  |  |
| Lymph node status | pN0 | 8 (24) | 0.413 | **0.000*** |
|  | pN1 | 11 (31) |  |  |
|  | pN2 | 11 (31) |  |  |
|  | pN3 | 3 (14) |  |  |
| Distant metastasis | M0 | 29 (83) |  |  |
|  | M1 | 6 (17) |  |  |
| Grading | Grade 1 | 3 (9) | 0.413 | 0.411 |
|  | Grade 2 | 19 (54) |  |  |
|  | Grade 3 | 13 (37) |  |  |
| Estrogen receptor status | ER- | 17 (40) | 0.482 | 0.214 |
|  | ER+ | 18 (60) |  |  |
| Progesterone receptor status | PR- | 17 (40) | 0.553 | 0.525 |
|  | PR+ | 19 (60) |  |  |
| Her2/neu status | Her2/neu- | 31 (89) | 0.665 | 0.153 |
|  | Her2/neu+ | 4 (11) |  |  |
| Grouped receptor status | ER, PR, Her2/neu- | 17 (49) | 0.289 | 0.095 |
|  | ER, PR,Her2/neu+ | 18 (51) |  |  |

**Table S1:** Association of PTK7 expression and patient´s clinico-pathological variables in primary tumors (BC) and lymph nodes (LN) by immunohistochemistry (IHC).
