# Supplementary material for: PTK 7 Is a Transforming Gene and Prognostic Marker for Breast Cancer and Nodal Metastasis Involvement
Source: PLoS One. 2014 Jan 7;9(1):e84472. doi: 10.1371/journal.pone.0084472 (PMC3883666; doi:10.1371/journal.pone.0084472)
Supplement: Text S1 — Immunohistochemistry. (DOCX) [file pone.0084472.s004.docx]

**Immunohistochemistry**

**Patients and Methods**

PTK7 IHC was performed with BC and LN (n=35) and three normal breast tissues of breast cancer patients (Table S1). Three micrometer slices of formalin-fixed, paraffin-embedded tumor tissues were deparaffinized in xylene and rehydrated through graded concentrations of ethanol to distilled water. Then endogenous peroxidase activity was blocked with 3% peroxide for 10 min and immunodetection of PTK7 was performed using the primary rabbit polyclonal anti- PTK7 antibody (Atlas Antibodies, Sweden) at a dilution of 1:200 (RT, 1h) and a heat antigen retrieval citrate buffer as a pretreatment (pH 6.0, 6 min). Sections were then incubated with the HRP-one step polymer (Zytomed Systems, Germany ), visualized using 3,3‘ Diaminobenzidin (DAB; Biogenex, USA) and then counterstained with hematoxylin (Merck, Germany). Finally the sections were dehydrated, cleared in xylene and mounted in Eukitt (VWR, Germany). A negative control (incubated without PTK7 antibody) was included every staining procedure to avoid unspecific staining.

Immunohistochemical evaluation of PTK7 staining was independently performed double blinded by two pathologists. The level of expression was scored according both the percentage of positive cells and the staining intensity of cytoplasm, that final scoring included four groups: < 10% stained tumor cells (score 0), >10% weak staining of PTK7 in tumor cells (score 1+), 10-30% intermediate staining of tumor cells (score 2+) and >30% high intensity of PTK7 staining in tumor cells (score 3+). For statistical analysis a binary score was performed and score 0/1+ were classified as negative, score 2+/3+ were ranked as positive.

**Results**

**IHC PTK7-expression and association with clinico-pathological features**

Positive staining of PTK7 was detected in 65.7% (23/35) of BC The intensity of PTK7 staining was stronger in TNBC (n=17), there we identified positivity of PTK7 in 88.2% (15/17) cases (P<0.000). Positive staining of PTK7 was also seen in 42.9% (15/35) of LN metastasis, whereas all cases of tumorfree LN (10/35) were PTK7 negative. As shown in Figure S2 PTK7 staining was predominantly located in the cytoplasm. Normal breast epithelial mostly showed weak PTK7 expression (score 0/1+ in 100%) (Figure S2 C). Comparing the association of IHC PTK7-expression in LN metastasis with clinico-pathological features, PTK7 occurred more frequently in premenopausal patients (P<0.001) with higher LN involvement (pN) (P< 0.000); this improved our findings of rtPCR. No further significant correlations between IHC PTK7 status and clinic-pathological features in BC or infiltrated LN were detected (Table S1). To evaluate the clinical impact of PTK7 in TNBC by IHC further investigations with an enlarged patient cohort are needed.
